# Pediatric Emergency Department Burn Discharge and Clinic Readiness: A Quality Improvement Project

**DOI:** 10.1097/pq9.0000000000000806

**Published:** 2025-04-16

**Authors:** Rachel Hatcliffe, Anne Ciriello, Elizabeth Murphy Waibel, Cindy Colson, Ashley White, Jennifer Fritzeen, Sarah Isbey

**Affiliations:** From the * Pediatric Emergency Department, Children’s National Hospital, Washington, D.C.; † Department of Trauma and Burn Surgery, Children’s National Hospital, Washington, D.C.

## Abstract

**Introduction:**

The shift to outpatient care for pediatric burn injuries has placed a greater responsibility on caregivers for wound care and follow-up planning. Nonadherence to burn care and follow-up appointments can lead to negative emotional and physical health outcomes. Both parental education and pain control with dressing changes are important factors for adherence to outpatient care. This single-center quality improvement project aimed to improve pediatric burn patients discharged from the emergency department with the correct instruction packet and the percentage of qualifying patients prescribed oxycodone for premedication for their initial clinic appointment.

**Methods:**

A multidisciplinary team retrospectively examined barriers using a fishbone diagram, developed a key driver diagram, and designed interventions, including updated custom instructions, printed discharge pamphlets, electronic medical record changes, enhanced e-prescribing access, linked International Classification of Diseases, Tenth Revision codes, targeted provider feedback, and education sessions. We tracked monthly data using statistical process control charts.

**Results:**

At baseline, 46% of patients received the correct discharge packet; following interventions, we observed a centerline shift to 78% with sustained improvement. Seventy percent of qualifying patients received an oxycodone prescription for premedication before clinic follow-up at baseline, and we saw a sustained baseline shift to 93% after interventions.

**Conclusions:**

Following multiple targeted interventions, there was a sustained improvement in the use of a custom burn discharge instruction packet and oxycodone prescriptions. Future research should examine the impact of discharge instructions and oxycodone prescriptions on the timeliness of outpatient appointment scheduling and pain scores.

## INTRODUCTION

In the United States in 2021, there were approximately 71,000 emergency department (ED) visits for nonfatal burn injuries to patients younger than 18 years of age, with over half of those occurring in patients younger than 6 years of age.^1^ While previously, many of these injuries may have necessitated admission, recent clinical practice has shifted toward ambulatory care in the treatment of burns with less than 10% total body surface area (TBSA) after initial ED evaluation.^2^ Continued care in the outpatient setting has many benefits, including decreasing unnecessary hospitalizations, reducing costs, and improving patient and parent satisfaction.^2,3^ Despite this overall trend, pediatric ED visits for burns demonstrate wide variability in follow-up and admission practices. Free-standing children’s hospitals have a lower admission rate of pediatric burn patients than combined pediatric and adult centers. The availability of a burn specialist consultation within an initial ED visit and comprehensive burn outpatient clinics at free-standing children’s hospitals may contribute to a heavier reliance on ambulatory management rather than admissions for continued care of pediatric patients with burn injuries. This possibility emphasizes the importance of ensuring safe, timely, and effective ambulatory care.^2,3^

Although the shift of burn care to the ambulatory setting can benefit patients, it also places a larger caregiving responsibility on families of pediatric patients. Successful patient outcomes depend on adequate parental education and pain control at home.^3,4^ Discharge instructions for burn injuries must include guidance on dressing changes, home pain control, signs and symptoms of infection, and scheduling outpatient visits. Incomplete instructions can lead to readmissions, unnecessary pain, and delay or lack of follow-up care.^5^ Given the long-term emotional and physical consequences of pediatric burn injuries, it is crucial to provide complete instructions with clear follow-up directives at ED discharge.^6^ Pain control is essential to a comprehensive ED discharge plan, as well as providing accurate and clear home care and follow-up instructions. Children with hand and foot burns, deep dermal partial-thickness injuries, and higher burn TBSA are at a greater risk of moderate-to-severe procedural pain during the first dressing change, which usually occurs in an outpatient clinic setting.^7^

## BACKGROUND AND SPECIFIC AIM

Before project initiation, providers in our outpatient burn clinic noted many patients were presenting for follow-up appointments without appropriate pain medication prescriptions or comprehensive discharge instructions. Providers also noted ED patients were unclear on how and when they should schedule outpatient appointments, leading to delays in care as well as inappropriate walk-in visits. Referred ED patients were also frequently unclear on when and how to dose premedication, including when a 1-time dose of oxycodone was prescribed by the ED to be given before the first dressing change in the outpatient burn clinic. In collaboration with ED providers, the team recognized the wide variability in the materials, instructions, and prescriptions that ED burn patients were receiving at the time of discharge at our institution and sought to standardize the process.

In this single-center quality improvement (QI) project at a regional pediatric burn center, our team sought to improve the percentage of patients given the correct custom discharge packets from 46% to 70% between March 2022 and December 2023, as well as to increase the percentage of qualifying patients prescribed a 1-time dose of oxycodone for clinic premedication from 72% to 90% between July 2021 and December 2023.

## METHODS

### Context

We completed this project in the pediatric ED at a large urban academic free-standing children’s hospital. This institution is a designated regional pediatric burn center serving a population of more than 1.4 million children in the DC-MD-VA metro area and surrounding counties.^8^ Combined, our main campus ED and satellite ED care for approximately 500 burn injuries annually, with 85% of treated burn patients in the ED discharged for outpatient follow-up. Our institution has a robust outpatient burn clinic with approximately 2,000 patient visits annually. Given the high number of visits requiring close ED follow-up, ED and burn teams rely on our discharge process to ensure appropriate, patient-centered continued care.

In April 2022, our multidisciplinary team of ED providers, burn team providers, and nursing staff created a custom, detailed burn discharge pamphlet (both available via paper copy and embedded in the electronic medical record [EMR]) with pertinent wound care and dressing information, return precautions, and contact information for on-call providers. The custom discharge instructions advise patients and their caregivers to use acetaminophen and ibuprofen every 6 hours as needed, including 30 minutes before dressing changes if indicated at home. The instructions provide specific on-call numbers depending on the time of day to be utilized if the pain does not improve with over-the-counter medications alone.

The EMR used at our institution is a Cerner product (Oracle Health, North Kansas City, Mo.). It includes multiple ED-specific order sets with embedded evidence-based decision-making tools and various custom and standard discharge instructions. Providers select the specific instructions they wish to provide their patients from multiple suggested options. An electronic order set within the EMR includes a prescription for a 1-time dose of oxycodone for eligible patients to be taken before their burn clinic appointment and dressing change.

**Fig. 1.**
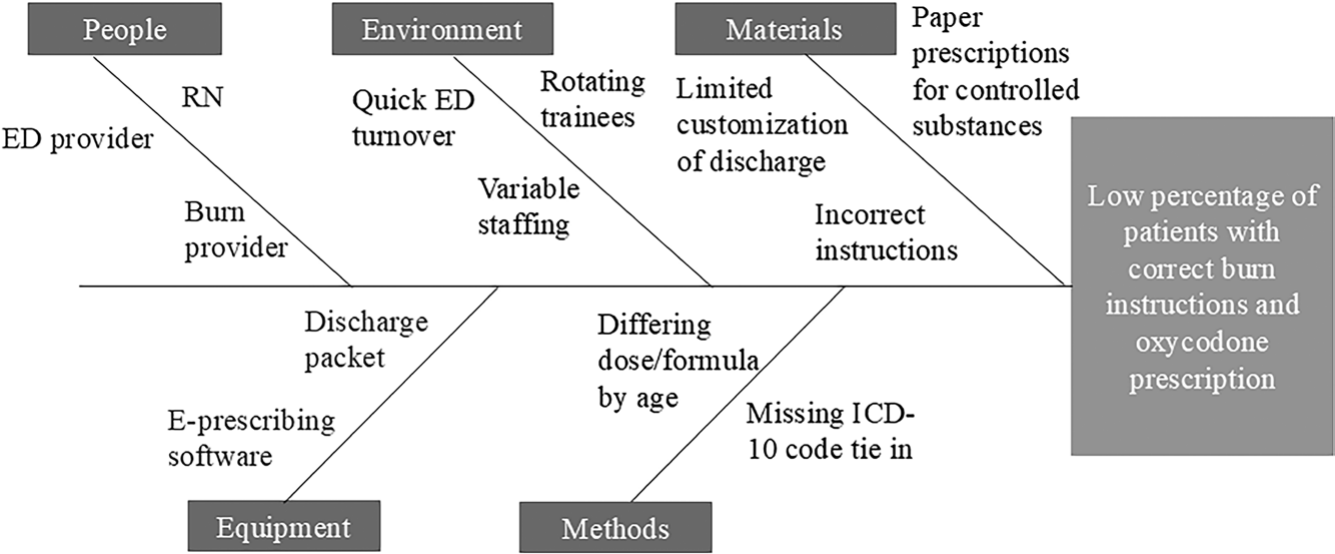
Fishbone diagram created by our multidisciplinary team to show possible root causes and areas of challenge regarding the discharge process, including selection of the correct instructions and discharge prescriptions.

**Fig. 2.**
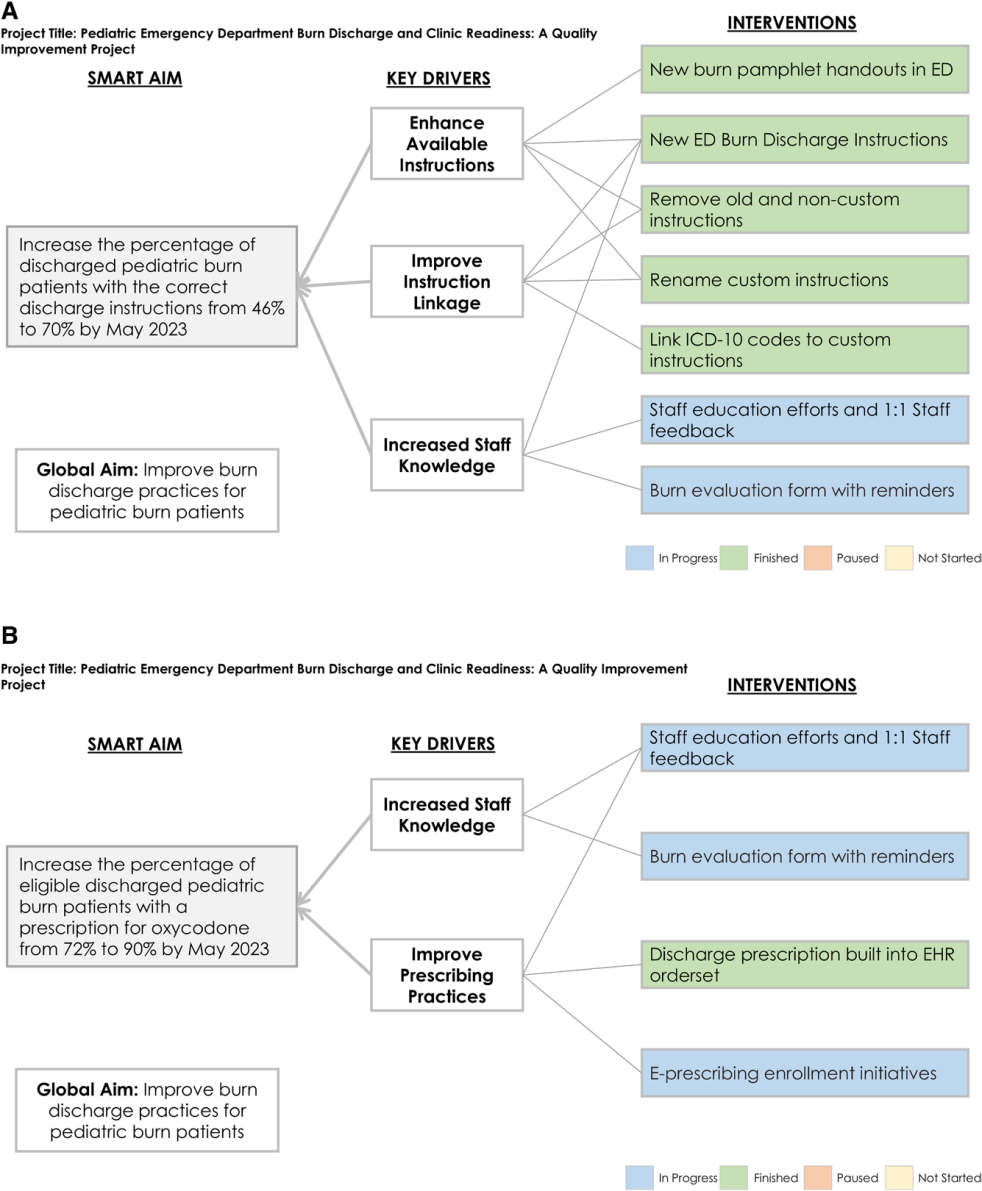
Key driver diagrams of burn discharge initiatives in the ED, including all pertinent interventions to discharge instructions (A) and discharge prescriptions (B).

We completed a current state analysis to understand why the correct discharge instructions were not included in ED discharge packets and why ED providers were not prescribing oxycodone to eligible patients. We created a fishbone diagram (Fig. 1) to understand the main barriers to appropriate discharge instructions and prescriptions. Subsequently, we used this information to create key driver diagrams (Fig. 2A and B) to identify interventions. This project focused on barriers to providers’ prescribing practices and correct custom instructions as part of a complete ED discharge for patients with second- or third-degree burn injuries discharged from the ED. This project was a QI initiative and did not require oversight by the institutional review board.

Eligible patients included any patient younger than 22 years of age discharged from the ED for a second- or third-degree burn-related diagnosis. All eligible patients required outpatient burn clinic follow-up. We excluded admitted patients and those with only first-degree burns as these injuries are generally self-limited and do not require specialized burn care follow-up. We limited oxycodone prescription inclusion criteria to patients older than 6 months of age with burn injuries equal to or greater than 1% TBSA and/or burns on the palms, face, or genitals. Burns on the palms, face, and genitals, while often less than 1% TBSA, require painful debridement, specialized dressings and close burn follow-up and thus were also included.

### Interventions

Interventions for discharge instructions selection and oxycodone prescribing included improving both electronic clinical decision support and staff education. In July 2021, the oxycodone discharge prescription became electronically incorporated into the burn care order set; however, at that time, providers were still required to print paper prescriptions for opioids. In April 2022, we merged 2 custom burn discharge instructions into a more comprehensive document in English and Spanish (**Supplemental Digital Content 1**, which shows burn care pamphlet in English, http://links.lww.com/PQ9/A650) (**Supplemental Digital Content 2**, which shows burn care pamphlet in Spanish, http://links.lww.com/PQ9/A651). We made this document available in the EMR and via paper copies in both EDs involved in our study. In July 2022, we linked updated discharge instructions to International Classification of Diseases, Tenth Revision codes related to burn injuries. From July to September 2022, we removed duplicated and outdated discharge instructions from the EMR. Additional EMR interventions included a burn evaluation alert that launches any time a provider initiates the burn order set, prompting providers through clinical decision-making rules (CDRs) for ED burn care and follow-up. Additionally, efforts to increase electronic prescriber enrollment occurred in December 2022 with a department shift toward electronic prescribing instead of the prior practice of printing paper prescriptions for controlled substances.

As our large academic medical center ED hosts rotating residents and medical students, multiple educational interventions were necessary to reach the most providers possible. Trainee educational interventions included lectures at weekly pediatric emergency medicine fellow conferences and burn care lectures during weekly rotating resident didactics beginning in September 2021. We presented educational sessions at a bimonthly ED staff meeting focused on improving oxycodone discharge prescriptions for eligible patients by reiterating criteria and updated order sets. Our team members initiated further targeted provider feedback via email for incorrect instructions and missing discharge prescriptions in October 2022. These interventions, along with intermittent educational sessions, continued into 2023.

### Analysis

The primary measures of this study were (1) correct electronic custom burn instructions provided to burn patients at discharge and (2) correct 1-time oxycodone prescriptions given to eligible patients. We calculated the provision of the desired custom discharge instructions using 29 months of data collected before initiation (November 2019 to March 2022). During the project, we tracked eligible patients monthly and performed a chart review to track percentages of correct discharge materials, prescription eligibility, and correct prescription route and dose. Additional paper copies of our burn discharge pamphlet were available in the ED for provider use as supplementary material for patients. However, we did not track this metric as tracking which patients received copies was difficult. For data purposes, we checked the electronic instructions that were printed and provided to patients at discharge because they are viewable in the patient’s EMR. We analyzed data using statistical process control charts. A centerline shift was determined using 8 or more consecutive data points above the mean for all p-charts. The t-chart special cause variation was analyzed using the rule of a single point outside the control limits. All SPC charts were created using QI Macros for Excel, version 2019.03 (Know Ware International, Inc., Denver, Colo.).

The balancing measures for this study included tracking oxycodone prescribing errors and unscheduled ED return rates. Given the aim of increasing oxycodone prescriptions for eligible patients, we tracked days between instances of incorrect oxycodone dosing throughout the study period to address possible adverse events or insufficient pain control. We analyzed these data using a t-chart. Unscheduled returns were tracked to ensure our new discharge instructions, while intending to be clearer and more comprehensive than prior iterations, did not lead to families returning to the ED instead of the intended follow-up in the burn clinic. Using a statistical process control chart, we tracked monthly unscheduled ED returns for patients presenting with burn injuries.

## RESULTS

**Fig. 3.**
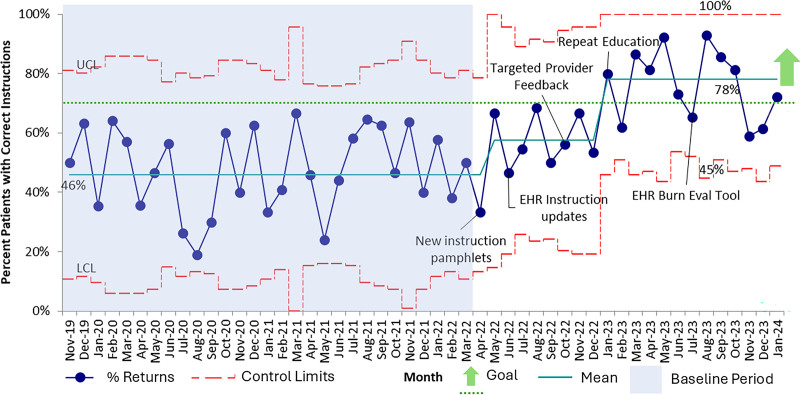
Statistical process control chart (p-chart) demonstrating monthly percentages of discharged pediatric ED patients receiving a custom burn instruction packet. EHR, electronic health record; LCL, lower control limit; UCL, upper control limit.

There were 3,036 unique visits for 2,895 patients from November 2019 to December 2023 for burn-related chief complaints. The admission rate for this time frame was 12% (n = 371 visits). Following burn pamphlet placement and streamlining of the EMR burn instructions, we saw a sustained shift in the centerline for the desired custom discharge instruction use from 46% to 58% from March 2022 to December 2022 (Fig. 3). The subsequent initiation of provider-level feedback on instruction use and multiple iterative education initiatives led to further improvement from 58% to 78%, with a second centerline shift by December 2023. These improvements were sustained through the end of data collection.

**Fig. 4.**
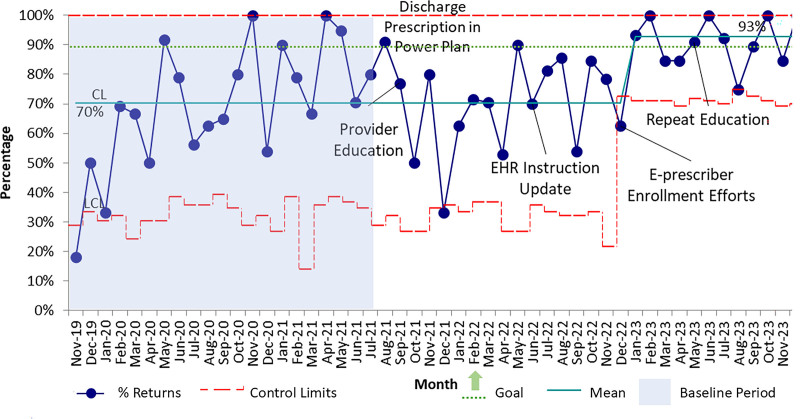
Statistical process control chart (p-chart) demonstrating monthly percentages of qualifying patients receiving a prescription for a 1-time dose of oxycodone. CL, centerline; EHR, electronic health record; UCL, upper control limit.

Seventy percent of eligible patients received an oxycodone prescription at discharge before any interventions (baseline period November 2019 to July 2021) (Fig. 4). There was no shift in prescription practices after placing a discharge prescription tool within the burn order set. However, after the initiation of provider education in September 2021, alongside an initiative to enroll all providers in electronic prescribing software for controlled substances, our team saw a centerline shift from 70% to 93% from December 2022 to December 2023. This improvement was sustained for the remainder of the study period.

**Fig. 5.**
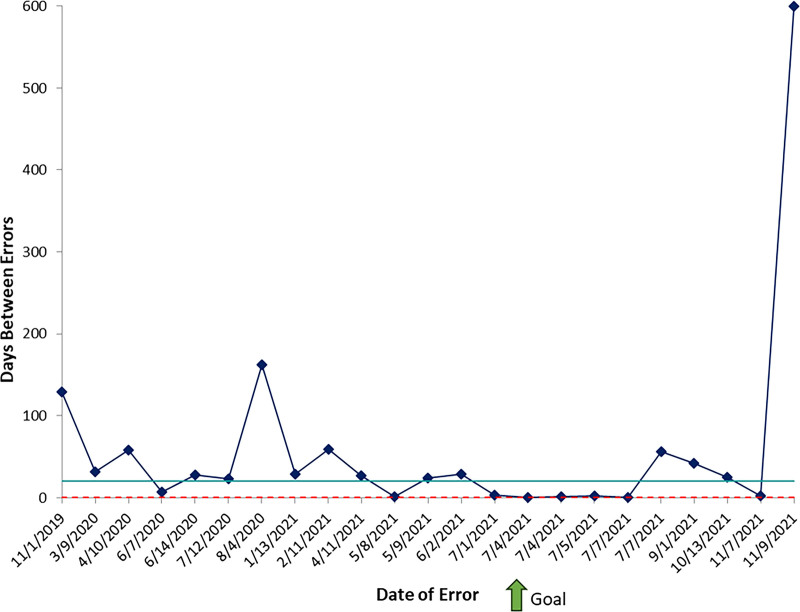
Statistical process chart (t-chart) demonstrating the days between errors in the dosing of discharge oxycodone prescriptions.

**Fig. 6.**
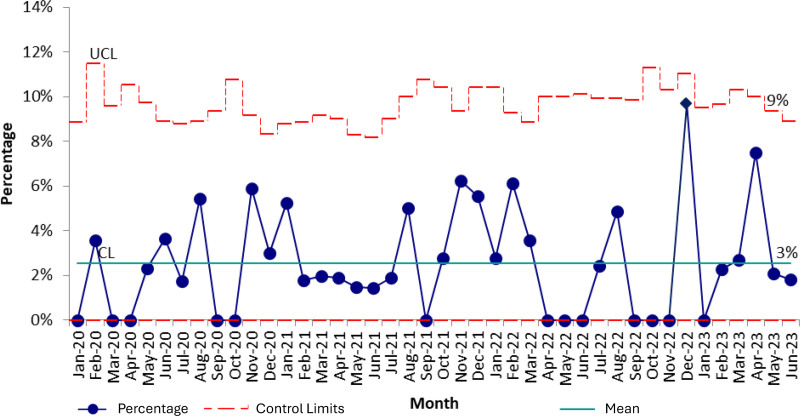
Statistical process chart (p-chart) demonstrating the monthly percentage of unscheduled returns for patients with burn injuries who are discharged from the pediatric ED. CL, centerline; UCL, upper control limi.

As balancing measures, we tracked oxycodone prescription errors and unscheduled returns or admissions of burn patients. Before study initiation, there were an average of 29 days between oxycodone prescription errors (baseline period November 2019 to July 2021) (Fig. 5). There was no special cause variation in prescription practices after placing a discharge prescription tool within the burn order set. However, the initiation of provider education in September 2021, alongside an initiative to enroll all providers in electronic prescribing software for controlled substances, led to special cause variation with a final data point outside the control limits and a final days-between count of 599 days at study end (no additional tracked error by study end). Regarding unscheduled ED returns, from January 2020 to June 2023, there was no change from baseline unscheduled returns (Fig. 6). Although the percentage of unscheduled returns was higher in December 2022, a chart review revealed 2 visits by the same patient with COVID-19-related complaints unrelated to her previous burn injury.

## DISCUSSION

Following multiple, diverse targeted interventions, our multidisciplinary team saw a sustained improvement in 2 discrete areas of interest: custom burn discharge instructions and the percentage of eligible patients discharged with oxycodone prescriptions for pain control before their initial dressing change in the burn clinic. Our results reinforce prior work improving discharge materials and outpatient referrals to patients in the pediatric ED treated for anaphylaxis, showing increased EMR efficiency and provider awareness leading to the most significant improvement.^9^

Even small burns can cause long-term physical and psychological consequences, including contractures, scarring, pruritis, anxiety, depression, posttraumatic stress, behavioral problems, as well as parental distress.^6,10–12^ Barriers to scheduling timely outpatient care, such as unclear paperwork, may lead to decreased follow-up despite parental interest in obtaining medical attention.^13^ Conversely, standardizing pediatric emergency room discharge processes has been associated with eliminating preventable discharge-related serious safety events.^14^

By ensuring the inclusion of accurate burn clinic contact information (including on-call information and a well-monitored burn care provider email), our team optimized the discharge process, which prior studies have shown increases outpatient follow-up and improves patient physical and emotional outcomes.^15,16^ Although we could not accurately track the distribution of the paper burn pamphlets, their presence in the ED may have served as a physical reminder to attach the correct custom instructions when discharging patients with burn injuries.

Challenges with ensuring correct instructions and oxycodone prescriptions for eligible patients included provider knowledge, time constraints in the pediatric ED, and competing discharge information within the EMR. Our interventions focused on refining the content and format of discharge instructions, removing extraneous content or contradictory information, and improving providers’ access to accurate instructions. We utilized CDRs within burn care order sets to provide direction for consultation during the ED visit and appropriate follow-up care and prescriptions. CDRs, such as those implemented above, are proven methods to establish baselines of care for specific disease processes and can aid providers in identifying appropriate treatment.^17^ Targeted provider feedback and education sessions reinforced the importance of providing oxycodone prescriptions at discharge. These sessions highlighted key changes made with the custom discharge instructions that provide improved directions for families.

Cleaning and debridement during dressing changes can also be significant sources of pain and anxiety for both caregivers and patients. Poor pain control can increase the risk of long-term emotional and psychological consequences.^18,19^ There is limited research examining the impact of electronic prescribing (e-prescribing) for opioids. However, e-prescribing, in general, in combination with EMR interventions such as pop-up alerts and incorporation into order sets, has contributed to a reduction in prescribing errors.^20^ E-prescribing improves pharmacy communication, reduces wait times and improves patient satisfaction.^21^ At our institution, facilitating oxycodone prescribing through order sets and increasing the number of providers with controlled substance e-prescribing privileges improved adherence to desired prescribing practices. Eliminating the step of having to retrieve a printed prescription and physically sign and hand the prescription to the discharging nurse or family may have contributed to provider compliance with opioid prescribing. Although this study focused on improving compliance with oxycodone prescriptions for eligible patients, future work could examine how preclinic pain medication administration impacts burn clinic visit duration and pain scores during appointments.

### Limitations

This study represents a single-center experience and interventions at a pediatric tertiary care center with a robust outpatient burn clinic. It, therefore, may not be easily extrapolated to community settings. Further expansion of these specific burn discharge practices to our region’s community and general ED setting may help improve follow-up for discharged pediatric burn patients. Although the percentage of correct discharge instructions assigned increased, targeted provider feedback is a significant time burden. With residents from multiple facilities rotating through the ED, it is challenging to capture a complete audience. During our education sessions on optimal burn discharges and our targeted feedback, we placed the ultimate responsibility for correct discharges on our permanent staff. This group includes attending physicians, fellows, and advanced practice providers. Targeted provider feedback may be more difficult in EDs with a heavier reliance on rotating trainees, locums, and part-time staff. Automated monthly feedback on compliance would make this intervention more sustainable in the long term.

This study was unable to capture barriers to patients obtaining prescriptions sent to pharmacies and did not track appropriate oxycodone administration before clinic appointments. Data regarding successful clinic follow-up, pain scores, and preclinic oxycodone dose administration were unavailable at the project initiation.

## FUTURE DIRECTIONS

Future research should examine the impact of improved discharge instructions and oxycodone prescriptions on outcome measures. These measures could include timeliness of outpatient appointment scheduling, clinic no-show rates, patient clinic pain scores, rates of prearrival medication administration, patient and family satisfaction, and long-term burn complications. A previous QI study at our institution focused on improving patient portal enrollment for discharged patients from the ED. It demonstrated sustained improvement with a follow-up nurse to educate families. A similar strategy could be used with our patient population to track patient satisfaction and schedule burn outpatient follow-up appointments.^22^

Previous research has shown that patients with lower socioeconomic status have higher rates of burn injuries as well as increased rates of nonadherence to follow-up plans.^23–25^ This study did not examine factors associated with outpatient compliance. Future studies could examine the demographic and encounter factors associated with follow-up rates.

## CONCLUSIONS

Through improving electronic clinical decision support and multipronged provider education efforts, we improved the provision of accurate and thorough discharge instructions and rates of oxycodone prescribing for pediatric patients with burn injuries discharged from the ED.

## ACKNOWLEDGMENTS

The author thank their colleagues in the ED and the Trauma/Burn Department for their ideas, collaboration, and participation, and Lauren Waterhouse for the contributions.

## Supplementary Material
