# Supplementary material for: Pediatric Emergency Department Burn Discharge and Clinic Readiness: A Quality Improvement Project
Source: Pediatr Qual Saf. 2025 Apr 16;10(3):e806. doi: 10.1097/pq9.0000000000000806 (PMC12002377; doi:10.1097/pq9.0000000000000806)
Supplement: Supplementary file 1 [file pqs-10-e806-s001.pdf]

CALL US IF YOUR CHILD...

- Has a **fever greater than 101.5°F, difficulty breathing or increased sleepiness**.
- Has **thick, bad smelling fluid** from the burn wound.
- Has **redness** around the burn.
- Has **bleeding** that continues after applying constant pressure for two minutes.
- Has a **dressing that will not stay in place**.
- Has a **dressing which has moved off the burn** and you can see the burned area.
- Has an outer **dressing that is completely wet**.
- **Will not eat or drink**. No urination or wet diapers in **8 hours**.
- Has **pain that does not go away** after using acetaminophen (Tylenol®) or ibuprofen (Motrin®).

AFTER THE BURN HEALS

Partial thickness burns and full thickness burns may take **several weeks to heal**. These burns may cause scarring (raised, thick, red skin).

- ☐ **Do not go out in the sun** between 10 a.m. - 2 p.m.
- ☐ **Use sunscreen** with an SPF of at least 25. Apply 30 minutes before going out into the sun. Reapply every 2 hours and after swimming or sweating.
- ☐ **Wearing a hat** will help protect your child from the sun if he/she has **burns to the face or neck**.
- ☐ **Massage therapy** can help lessen scar growth. Moisturizing can help with itching.
- ☐ **Compression garments** (stockings, gloves, etc.) are worn to help decrease scar growth by putting pressure on the burn.
- ☐ **Cica-Care® Silicone gel sheet** can be used for treating scars.

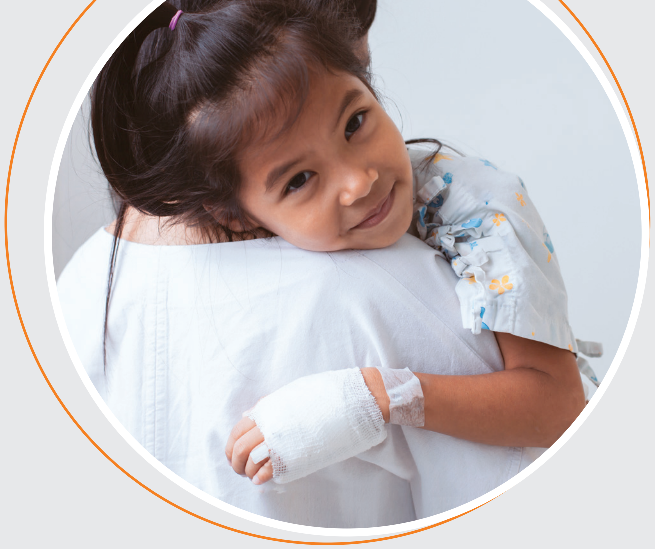

CONTACT INFORMATION

Weekdays 8 a.m.– 4 p.m.
202-476-5221
Burn and Trauma Nurse Practitioners
TraumaBurn@Childrensnational.org

Evenings and Weekends
202-476-5000
Ask for the senior pediatric surgical resident

Burn Clinic Appointment Line
202-476-2150

Burn Clinic Front Desk (Main Hospital)
Mon/Wed/Fri 8 a.m. - 12 p.m., and Wed 1 - 4 p.m.
202-476-2162

Burn Clinic Front Desk (Friendship Heights)
Wednesday 8 a.m. - 4p.m.
202-895-3860

Children's National
111 Michigan Ave NW
Washington, DC 20010
ChildrensNational.org

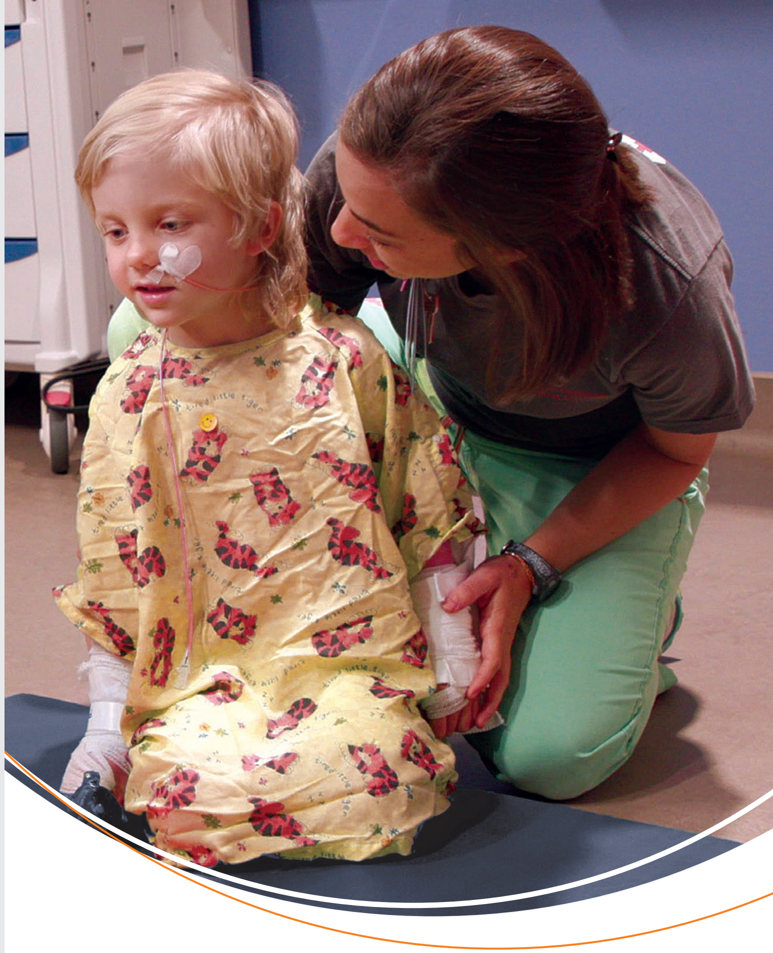

Burn Care

A GUIDE FOR PARENTS AND CAREGIVERS

Children's National

TYPES OF BURNS

There are three types of burns: **superficial, partial thickness, and full thickness**. The type of burn and how it was caused will decide the treatment.

- **SUPERFICIAL**
  Only the top layer of the skin called the epidermis is hurt. These burns are pink or red. First degree burns are painful, and dry (like a sunburn). There are no blisters. Heals in 3-7 days with no scarring.
- **PARTIAL THICKNESS**
  The epidermis (top layer of the skin) and the dermis (second layer of skin) are injured. Second degree burns are painful and will have blisters. Some second degree burns heal within 2-3 weeks without leaving a scar. Some partial thickness burns take 2-6 weeks to heal and may require a skin graft.
- **FULL THICKNESS**
  This burn includes all the layers of the skin (the entire dermis). These burns make the skin look shiny (waxy) and white. This is the most serious type of burn. Full thickness burns often require a skin graft and often take at least 3-6 weeks to heal.

After the Burn is a series of educational videos that will help you understand how to manage and care for your child's burn.
https://bit.ly/aftertheburn

LAYERS OF THE SKIN

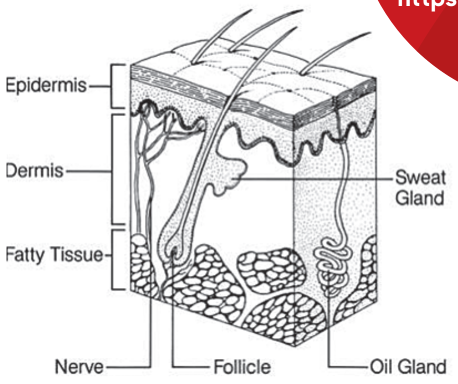

CHANGING THE DRESSING

☐ **Watch the video *At Home, Dressing Changes!*** This will guide you step by step on how to change the dressing and clean the burn:

https://bit.ly/aftertheburn

☐ **Long-acting burn dressing**
Do not clean the burn. Dressing to remain in place. Keep the dressing dry. Dressing change to be done in Burn Clinic at your next appointment.

☐ **Change the dressing and clean the burn once or twice a day.**

- You may give your child acetaminophen (Tylenol®) or ibuprofen (Motrin®) 30 minutes before you clean the burn.
- Soak your child's burn wound in warm, soapy water for 10 minutes. Younger children may soak in a tub and play while older children may prefer to take a shower.
- Wash your hands before cleaning your child's burn.
- Use a mild soap, such as Dove® or Johnson & Johnson® Baby Soap with a clean wet washcloth to gently wash the burn. This will remove all of the medication from the burn.
  - Be sure to wash away any dead skin.
  - The burn may bleed. Some bleeding is OK. If bleeding occurs, apply pressure to the burn.
- Gently pat the burn with a clean towel or gauze pad until it is dry.
- Using a tongue depressor or clean fingers, apply a thin layer of medication to the gauze. Cover the burn with the gauze. Make sure cream side touches the burn.
- Cover the burn with the gauze dressing and wrap with Kling®. Use tape to secure the dressing.

TREATMENT OF BURNS

☐ **Mepilex Ag® , Mepitel Ag®, or Acticoat® long-acting silver dressings**: Keep the dressing dry and covering the burn. No bathing, swimming or showering.

☐ **Bacitracin® Topical antibiotic**: Wash the burn twice daily and apply bacitracin.

☐ **Silvadene Cream 1%®, Sulfamylon®, Santyl®:** Apply a thin layer of cream to the gauze and place the gauze directly on the burn with the cream on the skin.

☐ **Xeroform**: Wash daily and apply the dressing to the skin.

☐ **Aquaphor® Moisturizer Healing ointment**: Clean twice a day and apply a thin layer.

☐ **Splint, stretching:** Helps to keep the skin from tightening up as the wound heals and provides comfort.

☐ **Moisturizing creams**: (Aquaphor®, Eucerin®, Aveeno®, Cocoa butter, Vasoline®) Apply a thin layer twice a day to healed skin

For pain: Give your child acetaminophen (Tylenol® or Tempra®) or ibuprofen (Motrin® or Advil®) every six hours if needed.
