# Supplementary material for: Pediatric Emergency Department Burn Discharge and Clinic Readiness: A Quality Improvement Project
Source: Pediatr Qual Saf. 2025 Apr 16;10(3):e806. doi: 10.1097/pq9.0000000000000806 (PMC12002377; doi:10.1097/pq9.0000000000000806)
Supplement: Supplementary file 2 [file pqs-10-e806-s002.pdf]

Llámenos si su hijo(a)...

- Tiene fiebre de más de 101.5°F, dificultad para respirar o mayor somnolencia.
- Tiene un líquido espeso y maloliente en la quemadura.
- Tiene enrojecimiento alrededor de la quemadura.
- Tiene sangrado que continua después de aplicar presión constante durante 2 minutos.
- Tiene un vendaje que no permanece en su lugar.
- Tiene un vendaje que se ha movido de la quemadura y usted puede ver el área quemada.
- Tiene un vendaje externo que está totalmente húmedo.
- No come ni toma líquidos. No ha orinado ni ha mojado el panal durante 8 horas.
- Tiene dolor que no se calma después de usar acetaminofén (Tylenol®) o ibuprofeno (Motrín®).

***Después de la quemadura** es una serie que incluye videos educativos acerca de todos los aspectos del cuidado de una quemadura. Si su hijo(a) está hospitalizado o ya está en casa, estos videos le ayudarán a entender cómo manejar y cuidar la quemadura de su hijo(a).*

Vaya a:

https://bit.ly/aftertheburn

Escanee el Código QR aquí

Después de que sana la quemadura

Las quemaduras de espesor parcial pueden tardar varias semanas en sanar. Estas quemaduras podrían causar cicatrices (piel abultada, gruesa y enrojecida).

No salga al sol entre las 10 a.m. - 2 p.m.

Use bloqueador solar con un SPF de al menos 25. Aplíquelo 30 minutos antes de salir al sol. Re aplíquelo cada 2 horas y después de nadar o sudar.

Usar un sombrero protegerá a su hijo(a) del sol si tiene quemaduras en la cara o el cuello.

La terapia de masaje puede reducir el crecimiento de cicatrices.

La hidratación ayuda a calmar la comezón.

Las prendas de compresión (medias, guantes, etc.) se usan para reducir el crecimiento de las cicatrices al poner presión a la quemadura.

Cica-Care® lámina de gel de silicona se puede usar para el tratamiento de las cicatrices.

Información de contacto

Entre semana 8 a.m.– 4 p.m.
202-476-5221
Enfermeras especializadas en quemaduras y traumatología
TraumaBurn@Childrensnational.org

Noches y fines de semana 202-476-5000
Pregunte por el residente de cirugía pediátrica principal

Línea de citas para la clínica de quemaduras
202-476-2150
Recepción de la clínica de quemaduras (Hospital Principal) lunes, miércoles y viernes- 8 a.m. - 12 p.m., y miércoles 1p.m. - 4 p.m. 202-476-2162

Recepción de la clínica de quemaduras (Friendship Heights) miércoles 8 a.m. - 4p.m.
202-895-3860

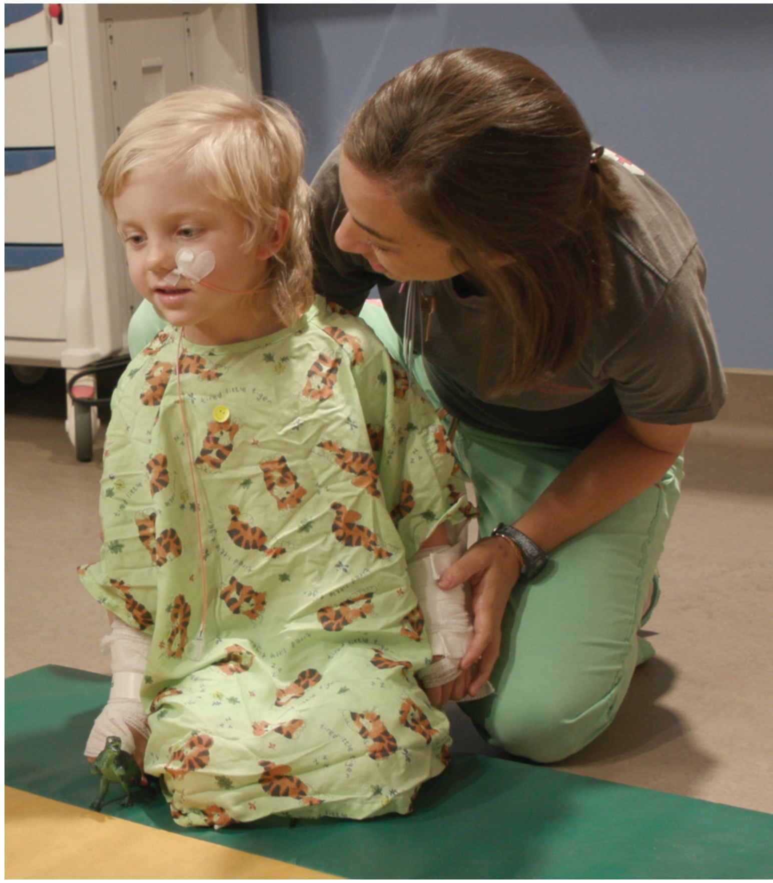

Cuidado de las quemaduras

Una **Guía** para Padres y Cuidadores

Children's National®

Tipos de quemaduras

Existen tres tipos de quemaduras: **superficial**, **espesor parcial**, **y espesor total**. El tipo de quemadura y qué la causo decidirá el tratamiento.

- Mire el video Understanding a Burn Injury (Para comprender una quemadura) enlace: **https://bit.ly/aftertheburn** para saber más acerca de la quemadura de su hijo(a).

- Superficial

Solo lastima la capa externa de la piel, llamada la epidermis. Estas quemaduras son rosadas o rojas. Las quemaduras de primer grado son dolorosas y secas (como las del sol). No hay ampollas. Sanan en 3-7 días sin dejar cicatriz.

- Espesor parcial

Se lastiman la epidermis (capa externa de la piel) y la dermis (la segunda capa de la piel). Las quemaduras de segundo grado son dolorosas y tienen ampollas. Algunas quemaduras de segundo grado sanan dentro de 2-3 semanas sin dejar cicatriz. Algunas quemaduras de espesor parcial sanan en 2-6 semanas y podrían requerir un injerto de piel.

- Espesor total

Estas quemaduras afectan todas las capas de la piel (toda la epidermis). Estas quemaduras hacen que la piel se vea brillante (cerosa) y blanca. Este es el tipo de quemadura más grave. Las quemaduras de espesor total con frecuencia requieren un injerto de piel y sanan en al menos 3-6 semanas.

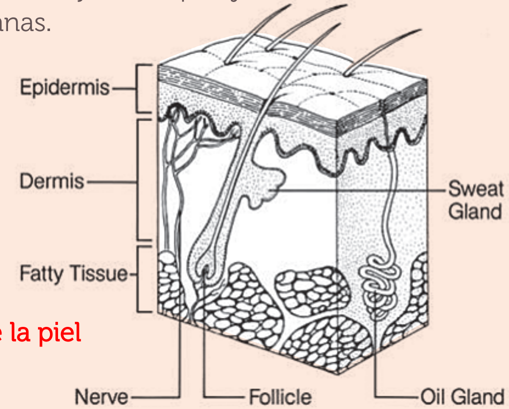

Capas de la piel

Para cambiar el vendaje

☐ **Vendaje para quemadura de acción prolongada**

No limpie la quemadura. El vendaje debe permanecer en su lugar. Mantenga el vendaje seco. El cambio de vendaje deberá realizarse en la Clínica de Quemaduras en su siguiente cita.

☐ **Cambie el vendaje y limpie la herida una o dos veces al día.**

- Le puede dar a su hijo(a) acetaminofén (Tylenol®) o ibuprofeno (Motrin®) 30 minutos antes de limpiar la quemadura.
- Remoje la quemadura de su hijo(a) en agua tibia y jabonosa durante 10 minutos. A los niños más pequeños los puede poner en la tina para jugar mientras que los niños más grandes pueden preferir ducharse.
- Lave sus manos antes de limpiar la quemadura de su hijo(a).
- Use un jabón suave, como Dove® o el jabón para bebé de Johnson & Johnson® con una toallita suave limpia para lavar con suavidad la quemadura. Esto removerá todo el medicamento de la quemadura.
  - Asegúrese de eliminar la piel muerta en el lavado.
  - La quemadura podría sangrar. Algo de sangrado está bien. Si hay sangrado, aplique presión a la quemadura.
- De palmaditas suavemente con una toallita o gasa limpia hasta que se seque.
- Usando un depresor lingual o con sus dedos limpios aplique una capa delgada de medicamento a la gasa. Cubra la herida con la gasa. Asegúrese que el lado de la crema toque la quemadura.
- Cubra la quemadura con el vendaje de gasa y envuelva con Kling®. Use cinta para fijar la venda

Tratamiento de quemaduras

☐ **Mepilex Ag® , Mepitel Ag®, or Acticoat® apósitos de acción prolongada con plata**

Mantenga el vendaje seco y que cubra la quemadura.
No se bañe, nade, ni se duche.

☐ **Bacitracin® antibiótico tópico.**
Lávela diariamente y aplique el apósito a la piel.

☐ **Crema Silvadene 1%®, Sulfamylon®, Santyl®**

Aplique una capa delgada de crema a la gasa y coloque la gasa directamente sobre la quemadura con la crema sobre la piel.

☐ **Xeroform**

Lávela diariamente y aplique el apósito a la piel.

☐ **Ungüento Aquaphor® hidratante curativo.**
Limpie dos veces al día y aplique una capa delgada.

☐ **Férula, estiramiento**

Ayuda a que la piel no se estire a medida que sana la herida y proporciona confort.

☐ **Cremas humectantes**

(Aquaphor®, Eucerin® , Aveeno® , manteca de cacao (Cocoa butter), Vasoline®)

Aplique una capa delgada 2 veces al día a la piel cicatrizada

Para el dolor: Dele a su hijo(a) acetaminofén (Tylenol® o Tempra®) o ibuprofeno (Motrin® o Advil®) cada 6 horas si es necesario.

Para cambiar el vendaje

☐ **Mire el video At Home, Dressing Changes (En casa, cambios de vendaje).**

Este video le guiará paso a paso en cómo cambiar un vendaje y limpiar la quemadura. https://bit.ly/aftertheburn
